# Effect of Acute Intermittent CPAP Depressurization during Sleep in Obese Patients

**DOI:** 10.1371/journal.pone.0146606

**Published:** 2016-01-05

**Authors:** Jonathan C. Jun, Dileep Unnikrishnan, Hartmut Schneider, Jason Kirkness, Alan R. Schwartz, Philip L. Smith, Vsevolod Y. Polotsky

**Affiliations:** Department of Medicine, Division of Pulmonary and Critical Care Medicine, Johns Hopkins University School of Medicine, Baltimore, MD, United States of America; Charité—Universitätsmedizin Berlin, GERMANY

## Abstract

**Background:**

Obstructive Sleep Apnea (OSA) describes intermittent collapse of the airway during sleep, for which continuous positive airway pressure (CPAP) is often prescribed for treatment. Prior studies suggest that discontinuation of CPAP leads to a gradual, rather than immediate return of baseline severity of OSA. The objective of this study was to determine the extent of OSA recurrence during short intervals of CPAP depressurization during sleep.

**Methods:**

Nine obese (BMI = 40.4 ± 3.5) subjects with severe OSA (AHI = 88.9 ± 6.8) adherent to CPAP were studied during one night in the sleep laboratory. Nasal CPAP was delivered at therapeutic (11.1 ± 0.6 cm H_2_0) or atmospheric pressure, in alternating fashion for 1-hour periods during the night. We compared sleep architecture and metrics of OSA during CPAP-on and CPAP-off periods.

**Results:**

8/9 subjects tolerated CPAP withdrawal. The average AHI during CPAP-on and CPAP-off periods was 3.6 ± 0.6 and 15.8 ± 3.6 respectively (p<0.05). The average 3% ODI during CPAP-on and CPAP-off was 4.7 ± 2 and 20.4 ± 4.7 respectively (p<0.05). CPAP depressurization also induced more awake (p<0.05) and stage N1 (p<0.01) sleep, and less stage REM (p<0.05) with a trend towards decreased stage N3 (p = 0.064).

**Conclusion:**

Acute intermittent depressurization of CPAP during sleep led to deterioration of sleep architecture but only partial re-emergence of OSA. These observations suggest carryover effects of CPAP.

## Introduction

Obstructive Sleep Apnea (OSA) is a common breathing disorder characterized by intermittent collapse of the airway during sleep, for which continuous positive airway pressure (CPAP) is the principal treatment. CPAP increases the intra-luminal pressure of the airway, overcoming the collapsing pressures of the surrounding tissues[1]. OSA is typically regarded as a chronic and persistent condition that does not remit without significant anatomical changes in weight or airway caliber. Patients are encouraged to wear CPAP continuously throughout sleep on a consistent basis. However, whether CPAP is necessarily “an everyday therapy” has been questioned[2]. When CPAP is discontinued, some studies suggest that OSA does not immediately revert to baseline severity within the first night of withdrawal[2–6]. These studies suggest that there may be carryover effects of CPAP that ameliorate the resumption of OSA.

Patients often use CPAP irregularly, including sporadic usage within a single night[7]. It is not currently known to what extent OSA recurs during these within-night interruptions of CPAP. To address this question, we induced hour-long CPAP depressurization episodes alternating with therapeutic pressure during sleep in patients with a history severe OSA and objective chronic CPAP adherence. We hypothesized that previously observed carryover effects of CPAP would manifest during these depressurization episodes.

## Materials and Methods

### Patient Selection

Patients were recruited from the sleep clinics at the Johns Hopkins and Johns Hopkins Bayview Sleep Disorders Center. This study was approved by Johns Hopkins IRB NA_00086830. All participants gave written consent for participation. Patients were recruited with severe sleep apnea and a good history of CPAP adherence. Specifically, Inclusion criteria included adults of all races ages 20–65 years old with a history of severe OSA (AHI>40 events/hour) documented within the preceding 4 years. They also needed clinical documentation of good adherence characterized by CPAP use for >70% of nights exceeding 4 hours in the previous month, or a clinic visit within the preceding year where the patient exhibited ≥3 months of equivalent adherence. A total of 9 subjects (7 men, 2 women) were recruited for the study. 1 subject was not able to tolerate the CPAP depressurizations and left after approximately 1 hour of sleep. Remaining subjects completed the protocol without difficulty and their results were included in the analysis. Exclusion criteria included uncontrolled hypertension with blood pressure > 160/100; history of congestive heart failure; insulin dependent diabetes; pregnancy; or commercial driver or pilot unable to refrain from duties the day following study. Some of the exclusions above were related to metabolic outcomes that are planned in separate analyses. The study was approved by the institutional review board of Johns Hopkins. All subjects gave informed consent prior to participation.

### Polysomnographic measurements

After informed consent, subjects reported to the clinical research unit at the Johns Hopkins Bayview Medical Center. Standard polysomnographic recording techniques were used with a full montage that included electroencephalograms (C_3_-A_2_, C_3_-O_1_, F_3_-A_2_), bilateral electro-oculograms, submental electromyogram, 3-lead electrocardiogram, and oxyhemoglobin saturation. Respiratory effort was measured by thoraco-abdominal movement assessed by mercury strain gauges. Leg movements were monitored by electrodes placed on the anterior tibialis muscles. Signals were recorded on a computer with RemLogic software. Transcutaneous CO_2_ monitoring (TCM4, Radiometer) was used to confirm that no hypoventilation during sleep occurred when CPAP pressure was lowered to atmosphere. Airflow was measured using a pneumotachograph (Hans Rudolph, model #4830). Nasal pressure was measured using a pressure transducer (Embla N7000, Medcare, Buffalo NY) from a side port in the nasal mask. Pressure was delivered to the nasal mask using a customized CPAP device (ResMed, Bella Vista, NSW Australia) capable of rapid remote controlled pressure adjustments.

### CPAP depressurization protocol

**Fig 1:**
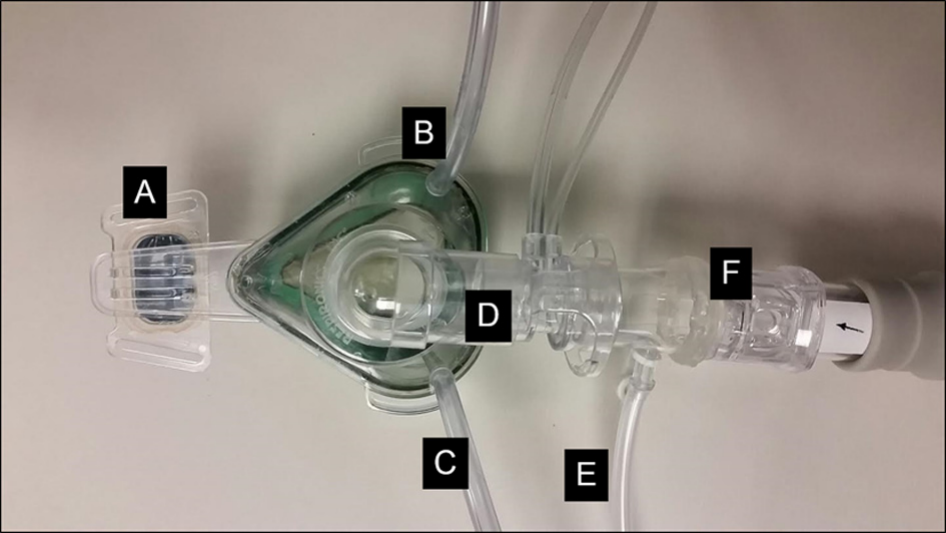
CPAP study circuit. Subjects were provided with CPAP capable of alternating rapidly between therapeutic pressure or atmospheric pressure. A: Nasal mask, B: Mask feedback pressure monitoring (used to maintain nasal pressure at target level), C: cannula delivering 5 L/min compressed air during CPAP-off periods, D: Pneumotachograph, E: Nasal pressure recorder, F: One way anti-asphyxia valve.

Subjects slept with nasal CPAP (ResMed Mirage Vista mask). The nasal mask was equipped with an anti-asphyxia valve which prevented CO_2_ accumulation during CPAP-off periods. A bias flow of air at 5L/min was also provided to the nasal mask during CPAP-off periods to minimize dead space breathing. We confirmed that this flow of air was not perceptible by subjects, and did not lead to any detectable changes in mask pressure (as measured by pneumotachograph from port B in Fig 1) or in gas exchange.

The CPAP setup is shown in Fig 1. From 22:00 until 23:00, the subject’s prescribed CPAP pressure was delivered to prevent obstructive hypopneas and apneas. If obstructive events were still noted, CPAP could be titrated upwards (this was not necessary in any of the patients studied). Thereafter, CPAP pressure was dropped to atmospheric pressure for 1 hour, every other hour. This was achieved in 2 cm H_2_0 increments every 3 breaths to avoid sudden shifts in pressure or noise from rapid closure of the anti-asphyxia valve.

### Sleep Scoring

Sleep was staged and respiratory events scored according to American Academy of Sleep Medicine guidelines [8]. Apneas were considered present in the absence of flow for >10 seconds. Obstructive apneas were considered present when apneas were associated with positive strain gauge deflections indicating thoracic movement. Apneas were considered central in origin when cessation of airflow was not accompanied by thoracic movement as measured by the strain gauge. Mixed apneas were defined by episodes of no air movement resulting from central apnea followed by obstruction. Hypopneas were scored for reductions in airflow of 30% lasting >10 seconds accompanied by either an arousal or fall in oxyhemoglobin saturation of 3% or greater. The scoring of the previously performed baseline clinical studies, and the current research study were performed using the same criteria, and reviewed by the same sleep physician.

### Statistical Analysis

Each study was divided into 8 hour-long blocks, each with its own sleep staging and tabulation of respiratory events including the apnea hypopnea index (AHI), 3% oxygen desaturation index (ODI), average SpO_2_ nadir, arousal index (AI) and respiratory arousal index (RAI). All indices were calculated based on time asleep during each hour. We combined the data from the 4 CPAP-on hours and the 4 CPAP off-hours to and compared the two groups using a Wilcoxon matched-pair signed rank test. A similar comparison was made for CPAP-on and CPAP-off periods (hour 1 vs 2, hour 3 vs 4, etc.) In all figures, the mean and standard error of the mean are depicted. Additional analyses were performed to examine effects of time, CPAP state, or their interaction on AHI and sleep architecture parameters. A two-way analysis of variance (ANOVA) with repeated measures was used for this analysis. Calculations were performed in GraphPad Prism 6.0 (GraphPad Software, La Jolla CA). Comparisons were considered significant at the p<0.05 level.

## Results

Demographics of study subjects are shown in *Table 1.* Most of the study subjects were middle aged (48.1 ± 3.3 years) morbidly obese (BMI = 40.4 ± 3.5) men. At the time of study entry, 2.5 ± 0.4 years had elapsed since the initial clinical PSG when OSA was diagnosed. Subjects gained 5.3 ± 3.6 kg during this time interval. Baseline clinical PSG results are shown in *Table 2*. Subjects BA and DR underwent split-night studies while the remaining subjects underwent full night PSG.

**Table 1:** Clinical characteristics of study group.

Subject	Sex	Age (yr)	Weight (kg)	Ht (cm)	BMI	Years since PSG	Δweight (kg)
1	M	59.6	99.8	175.3	32.6	3.1	-1.0
2	F	35.1	133.4	162.6	48.7	1.8	10.6
3	M	43.8	136.5	180.3	38.6	3.6	24.4
4	M	36.3	110.7	167.6	39.1	0.9	2.0
5	M	49.6	122.0	182.9	35.8	1.1	5.0
6	M	57.8	120.2	182.9	34.6	3.5	9.6
7	F	47.2	171.0	165.1	61.3	3.4	3.2
8	M	55.2	92.1	172.7	32.6	2.6	-11.4
AVERAGE		48.1	123.2	173.7	40.4	2.5	5.3
STD ERR		3.3	8.7	2.8	3.5	0.4	3.6

**Table 2:** Sleep architecture during clinical PSG at time of diagnosis.

Subject	Sleep time (min)	Sleep efficiency (%)	%N1	%N2	%N3	%REM
1	346.5	91.8	15.3	54.7	26.6	3.5
2	115.9	97.0	19.8	61.7	2.5	16.0
3	436.8	96.4	8.7	43.6	24.2	23.5
4	360.0	91.4	30.6	51.7	0.0	17.8
5	79.5	49.3	99.4	0.6	0.0	0.0
6	61.0	29.7	72.1	27.9	0.0	0.0
7	308.8	90.8	26.2	56.0	0.0	17.7
8	590.5	95.2	11.6	51.1	10.2	27.0
AVERAGE	287.4	80.2	32.9	35.7	7.9	13.2
STD ERR	66.5	9.1	12.2	8.3	4.0	3.8

Tables 2 and 3 show the baseline sleep architecture and sleep apnea characteristics. Sleep efficiency was good (80.2 ± 9.1%) with relatively high levels of stage N1 sleep and low levels of stage N3 and REM sleep. All subjects had severe, predominantly obstructive (83.9% of events) sleep apnea with an average AHI of 88.9 ± 6.8. The apnea index was 54.5 ± 11.7 and the hypopnea index was 34.3 ± 12.4.

**Table 3:** OSA Characteristics at time of diagnosis.

Subject	AHI	NREM AHI	REM AHI	%Obstructive	Supine AHI	Non-Supine AHI	SpO2 average nadir (%)	SpO2 min (%)	3% ODI	CPAP Pressure (cm H20)
1	82.1	81.9	88.2	60.0	82.1	N/A	91.8	87.0	66.5	8
2	93.3	94.7	85.7	100.0	30.5	114	85.7	93.3	63	12
3	63.0	58.4	77.8	72.4	74.1	60.1	88.4	79.2	65	12
4	114.6	120.8	86.4	100.0	117.6	N/A	81.7	67.2	91	10
5	100.0	100.0	N/A	100.0	114.3	93.7	91.3	87.0	82	10
6	68.8	68.8	N/A	55.7	44.4	88.4	90.4	87.0	59	11
7	111.4	120.0	71.3	100.0	111.4	N/A	80.9	72.1	111	14
8	77.6	76.0	81.7	83.1	93.4	68.2	90.2	82.3	68.0	12
AVERAGE	88.9	90.1	81.9	83.9	83.5	84.9	87.6	81.9	75.7	11.1
ST ERR	6.8	8.1	2.6	6.7	11.5	9.6	1.5	3.1	6.3	0.6

As a group, based on the data available, there was neither an effect of REM sleep nor body position on the severity of OSA. However, no periods of non-supine sleep were captured in 3 subjects, and no periods of REM were captured in 3 others. The average SpO_2_ nadir during respiratory events was 87.5% and the mean of the lowest SpO_2_ nadir was 81.9%. All subjects were accustomed to nasal CPAP masks at home. The mean therapeutic CPAP pressure used by subjects was 11.1 ± 0.6 cm H_2_0. Objective adherence monitoring demonstrated that subjects used CPAP on 97.0 ± 1.5% of nights. Four hours of CPAP use/night was exceeded on 86.5 ± 6.1% of nights. In 7/8 subjects, residual AHI data were available from their CPAP adherence card, with an average of 1.5 ± 0.8 events/hour.

**Fig 2:**
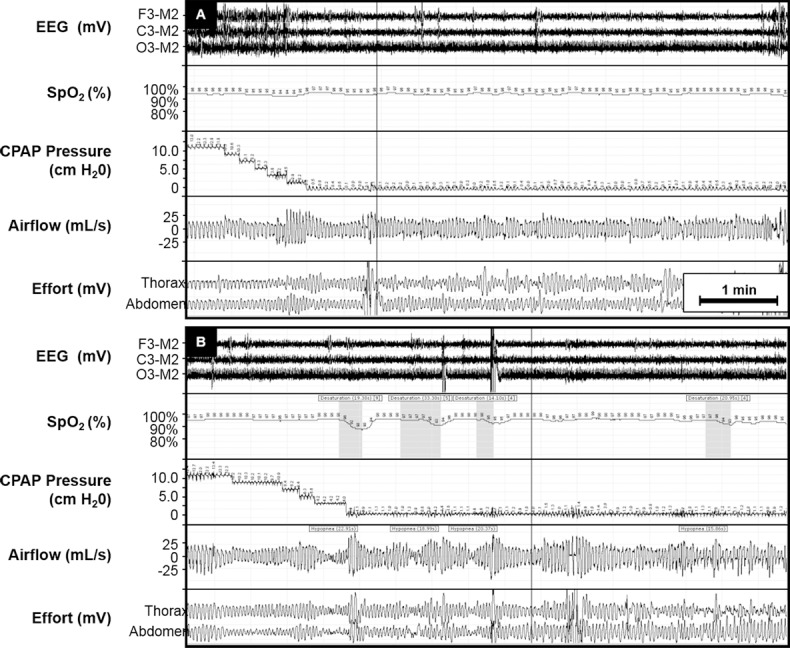
Representative tracing of CPAP depressurization during sleep in a subject with previous diagnosis of severe OSA. An approximately 7 minute period is depicted. (A) Rapid reduction in nasal pressure did not lead elicit significant obstructive events. In the same subject, a different period of CPAP depressurization (B) induced hypopneas.

**Fig 3:**
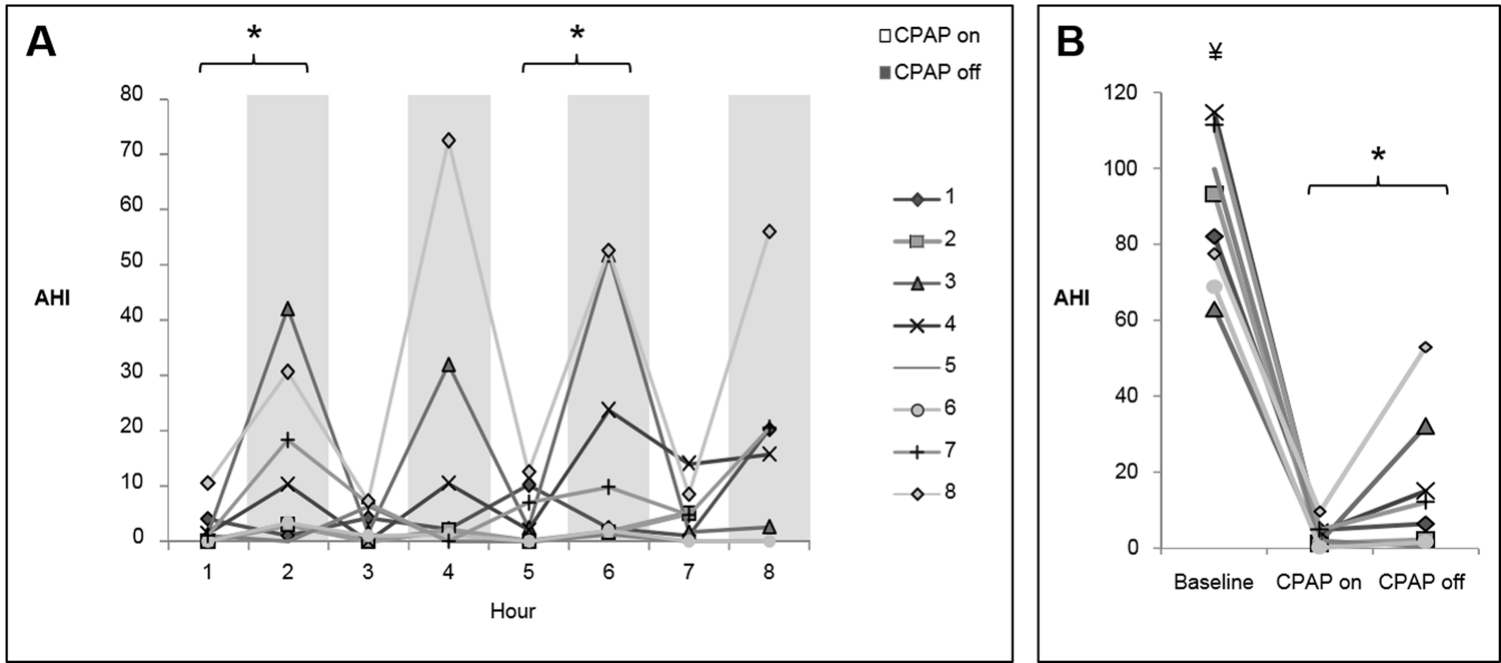
Extent of OSA recurrence as measured by AHI during CPAP withdrawal. (A) AHI across the night during alternating periods of therapeutic or atmospheric CPAP (*p<0.01 CPAP-on vs. CPAP-off). (B) Comparison of the baseline AHI, and the average AHI during CPAP-on and CPAP-off periods (*p<0.05 CPAP-on vs. CPAP-off; ¥p<0.001 vs. CPAP-on and CPAP-off).

**Fig 4:**
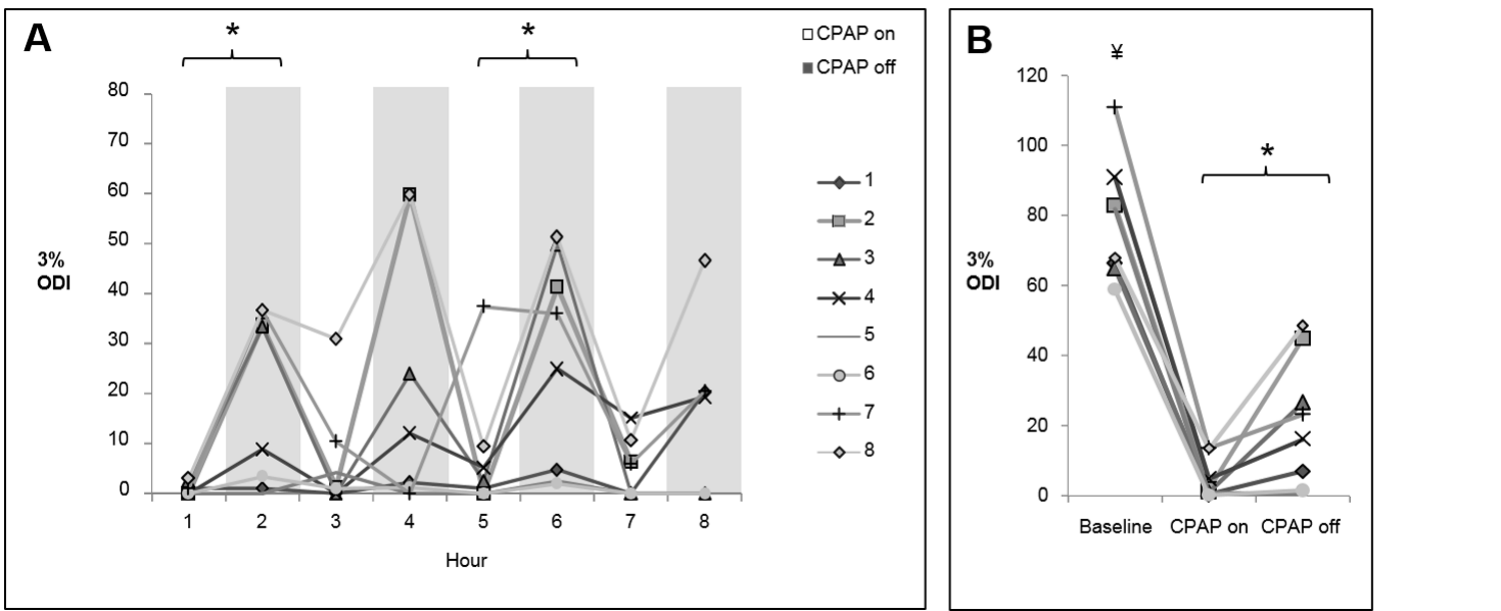
Extent of OSA recurrence as measured by ODI during CPAP withdrawal. (A) ODI across the night during alternating periods of therapeutic or atmospheric CPAP (*p<0.01 CPAP-on vs. CPAP-off). (B) Comparison of the baseline ODI, and the average ODI during CPAP-on and CPAP-off periods (*p<0.05 CPAP-on vs. CPAP-off; ¥p<0.001 vs. CPAP-on and CPAP-off).

In terms of the responses to CPAP depressurization, there was significant heterogeneity within subjects and between subjects. Fig 2 is provided as an example of these variable responses, sometimes resulting in minimal evidence of flow-limited breathing (Fig 2A), and sometimes resulting in recurrent hypopneas (Fig 2B). There were no detectable changes in transcutaneous CO_2_ during transitions between CPAP pressure (S1 Fig). OSA recurred in many subjects (Fig 3), but to a considerably milder extent than at time of diagnosis. The average AHI during CPAP-on and CPAP-off periods was 3.6 ± 0.6 and 15.8 ± 3.6 respectively (Fig 3B, p<0.05). The baseline AHI was significantly higher than both the CPAP-on AHI (p<0.001) and the CPAP-off AHI (p<0.05). As shown in Fig 4, a similar pattern was observed for the 3% ODI; the average 3% ODI during CPAP-on and CPAP-off was 4.7 ± 2 and 20.4 ± 4.7 respectively. Baseline ODI was significantly higher than both the CPAP-on ODI and the CPAP-off ODI (Fig 4B, p<0.001). The majority of respiratory events occurred during NREM sleep, since most subjects exhibited minimal REM sleep during CPAP off (S2 Fig). In a few instances, respiratory events occurred with CPAP on. Subject 7 (hour 5) and subject 8 (hour 3) experienced central apneas reflected in the ODI. Subject 4 (hour 7) had mild OSA while on CPAP, with an AHI of 15 during REM sleep. Otherwise, CPAP delivery effectively controlled sleep apnea. The effect of CPAP withdrawal on AHI was not influenced by elapsed sleep time (sleep hour), as evidenced by a relatively consistent pattern of OSA recurrence throughout the night (Fig 3). Two-way repeated measures ANOVA confirmed that time did not independently affect AHI (p = 0.76), nor was there a significant CPAP status x time interaction (p = 0.94).

Supine sleep is known to worsen OSA in some individuals. Therefore, we next examined whether changes in body position could have influenced the recurrence of OSA. Six of the 8 patients slept supine for the entire night. Subjects 2 and 7 spent 53.4% and 23% of the night in supine sleep, respectively. Subject 2 slept supine until 5th hour of sleep, then slept on his right side. The AHI was minimal regardless of body position. In subject 7, the patient spent 71 minutes of sleep in the supine position, of which 29 minutes were spent off CPAP. No apneas or hypopneas occurred during this period. Thus, while this data set is small, there was no evidence that attenuation of the AHI during CPAP withdrawal was related to non-supine sleep.

**Fig 5:**
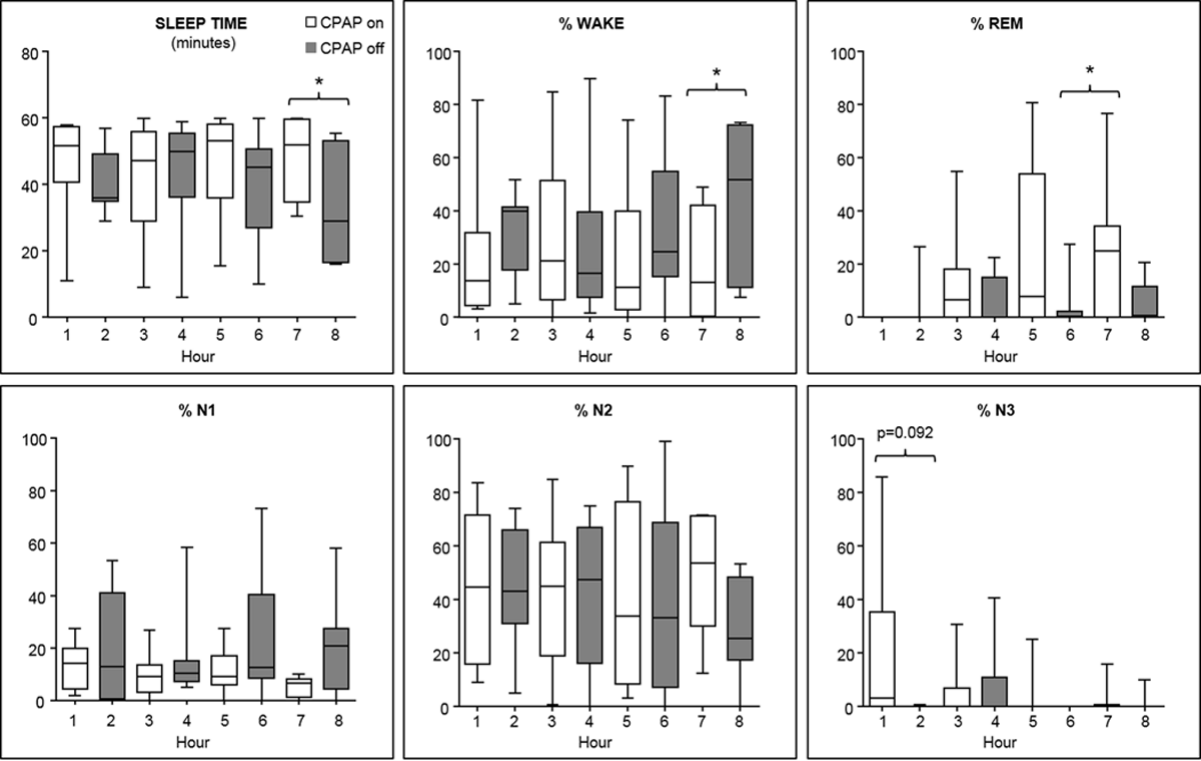
Effects of CPAP depressurization on sleep architecture across the night. Overall, CPAP depressurization reduced sleep time, increased the proportion of time spent in stage wake and N1, and reduced the proportion of time spent in stage REM sleep (p<0.05 for each comparison). More specifically, sleep efficiency was reduced during the fourth period of CPAP depressurization, as shown by both reduction in sleep time and increase in %wake. There was also reduced REM during the third period of CPAP depressurization (*p<0.05) and a trend towards reduced N3 sleep during the first CPAP depressurization. Median, interquartile range (box), and min/max (whiskers) are shown in each panel.

CPAP depressurization significantly affected sleep architecture throughout the night (Fig 5). Overall, CPAP withdrawal caused more %wake (p<0.05), %stage N1 (p<0.05), less %REM (p<0.05) and a trend towards decreased %N3 sleep (p = 0.064). There were also specific periods of sleep where CPAP withdrawal reduced sleep time, increased %wake, and reduced REM sleep, as indicated by the bracketed periods in Fig 5. There was no significant CPAP status x time interaction for any of these sleep parameters. However, there was a gradual increase in %REM and decrease in N3 sleep over the course of the night apparent only in the CPAP-on state. Accordingly, there were trend interactions for CPAP status x time for %REM (p = 0.11) and %N3 (p = 0.10) sleep. Thus, CPAP depressurization led to a deterioration of sleep architecture and a mild recrudescence of OSA.

## Discussion

In this study we found that intermittent depressurization of CPAP led to the emergence of mild OSA in CPAP-adherent obese subjects previously diagnosed with severe OSA. The degree to which OSA re-emerged varied considerably, but all 8 of the subjects analyzed exhibited milder event rates than at time of diagnosis. CPAP depressurization also altered sleep architecture, leading to more periods of wakefulness and stage N1 sleep and decreases in REM sleep. This, to our knowledge, is the first demonstration that intermittent CPAP depressurization does not lead to full return of baseline OSA in previously adherent CPAP patients.

CPAP withdraw has been explored in several other studies, ranging in duration from one night to 2 weeks of cessation. Most studies report the recurrence of OSA less severe than at the time of initial diagnosis [2–6]. For example, Kribbs *et al* showed that one night of CPAP withdrawal led to an respiratory disturbance index of 34, as compared to a pre-treatment frequency of 57[6]. Yang *et al* showed a return to baseline AHI on the first night of CPAP withdrawal, but a lesser degree of hypoxemia which ultimately returned to baseline severity after one week[5]. A randomized study comparing 2 weeks of CPAP withdrawal to CPAP continuation showed a rapid recurrence of OSA approaching the pre-treatment AHI within a single night of withdrawal, but an attenuated ODI until the subsequent night[3]. In a more recent study, OSA was found to not recur at all in 29% of subjects after 4 nights of CPAP withdrawal, particularly in subjects with less severe OSA and smaller body habitus[2]. Hence, this study is concordant with other studies showing mitigated OSA during acute CPAP withdrawal.

What factors might have moderated the return of OSA in the present study? An apparent improvement in OSA might merely reflect the known inter-night variability of OSA, which may lead to misclassification at low ranges of AHI [9–11]. This inherent variability is unlikely to account for the marked attenuation seen in severe OSA. There is also no reason to suspect spontaneous remission of OSA in subjects with increased age and weight. Nevertheless, the very severe baseline OSA status of these patients may have contributed to a regression towards a mean lowering of the AHI. Next, CPAP depressurization adversely affected sleep architecture, leading to increased stage wake and N1 and loss of REM sleep. In particular, reduced REM sleep might attenuate the overall severity of OSA. However, all of the subjects in this study demonstrated severe OSA in NREM sleep, and most did not have significant worsening in REM sleep during their baseline study. Furthermore, sleep instability with frequent arousals usually aggravates, rather than attenuates, OSA [12]. Still, we cannot rule out the possibility that at least part of the “protective” effect of CPAP depressurization was an inhibition of REM sleep. Finally, although great care was taken to minimize rebreathing and respiratory dead space in the CPAP circuit, it is possible that subtle CO_2_ accumulation or a slight increase in resistive breathing had a stabilizing effect during CPAP depressurization. Our results are qualitatively similar to above studies where CPAP was completely withdrawn, or delivered via mask in a subtherapeutic manner[3]. Taken together, we suspect that our findings are another manifestation of previously described carryover effects of CPAP. OSA has been proposed to be aggravated by factors such as edema resulting from snoring or apnea itself[13]; rostral fluid shifts during recumbent sleep[14], compromised airway sensory afferent reflexes[15]; or genioglossus muscle fatigue[16]. An enduring effect of CPAP might be expected if there is reversal of, or respite from these phenomena. An anatomical remodeling effect of CPAP was demonstrated in some studies after chronic CPAP use using magnetic resonance imaging [17] or acoustic phayngometry[18]. CPAP may also reduce the loop gain of the respiratory control system, blunting ventilatory responses that propagate respiratory instability[19,20]. We suspect that the substantial reduction in AHI observed in this study was related to the relatively brief periods of CPAP withdrawal, during which carryover effects of CPAP were still robust.

The primary strength of this study was the remote-controlled CPAP depressurization, without manipulating the mask or interacting with the patient in any other manner. This strategy captures the respiratory physiology of CPAP interruption, and overcomes the variability of sleep and OSA across different nights. However, our findings should be applied with several caveats in mind. First, we studied patients highly adherent to CPAP for years. The minimum duration and consistency of antecedent CPAP use to safely interrupt CPAP is not clear. It is also not certain whether preserved breathing would have continued beyond the one-hour periods studied. Secondly, this was a relatively small sample of predominantly male patients with morbid obesity and severe sleep apnea. OSA is now understood to be a heterogeneous disorder with variable causes and clinical manifestations[12]. Therefore, effects of CPAP depressurization could differ, especially in female and/or non-obese patients. Third, the natural behavior of OSA when CPAP is fully removed may differ from what was achieved by this research protocol. Fourth, we lacked the sample size necessary to draw definitive conclusions about interactions between CPAP depressurization, sleep stage, and body position. Fifth, we used a protocol in which CPAP pressure was delivered in the same order in each patient. This strategy allowed for titration of CPAP pressure, in case adjustments were necessary; and to facilitate sleep onset in these CPAP-adherent patients. It is possible that randomizing the order of CPAP pressure delivery (e.g. starting the night off CPAP, then delivering therapeutic pressure after one hour) could have led to different results. It would also be of interest to determine whether our findings apply to the common clinical practice of patients starting the night with CPAP on, but removing it hours later. A dedicated study of CPAP withdrawal midway through the night would be necessary to answer this question. Nonetheless, our findings corroborate with previous studies indicating that antecedent CPAP use mitigates recurrence of OSA.

There are two important clinical implications of this study. First, patients are currently considered to be “adherent” to CPAP if they use it for >4 hours per night 70% of the time. However, our findings suggest that efficacy of CPAP may be underestimated by assuming a linear relation with hours of use per night, particularly if CPAP is worn intermittently. Although a subset of patients may manifest minimal OSA during CPAP interruption, the factors that predict this phenotype will require further study. Second, CPAP interruptions alter sleep architecture. Patients should be encouraged to use CPAP in a continuous manner to optimize sleep architecture.

## Supporting Information

S1 Fig Transcutaneous CO_2_ measurements during the night according to CPAP status. (TIF)

S2 Fig AHI during NREM and REM sleep according to CPAP status during the night. (TIF)
