# Supplementary figures and images for: Effect of Acute Intermittent CPAP Depressurization during Sleep in Obese Patients
Source: PLoS One. 2016 Jan 5;11(1):e0146606. doi: 10.1371/journal.pone.0146606 (PMC4701426; doi:10.1371/journal.pone.0146606)

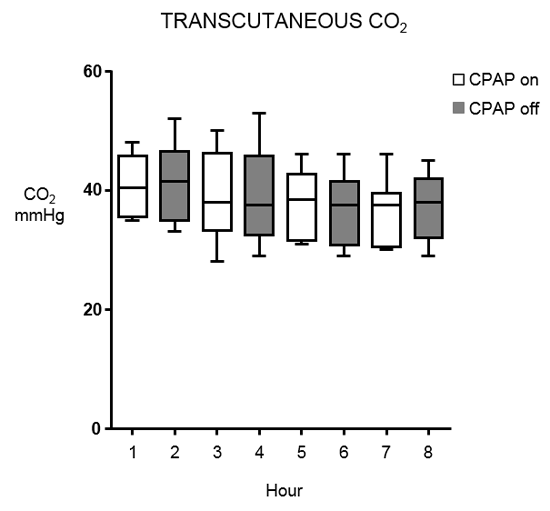

Supplement: S1 Fig — (TIF) [file pone.0146606.s001.tif]

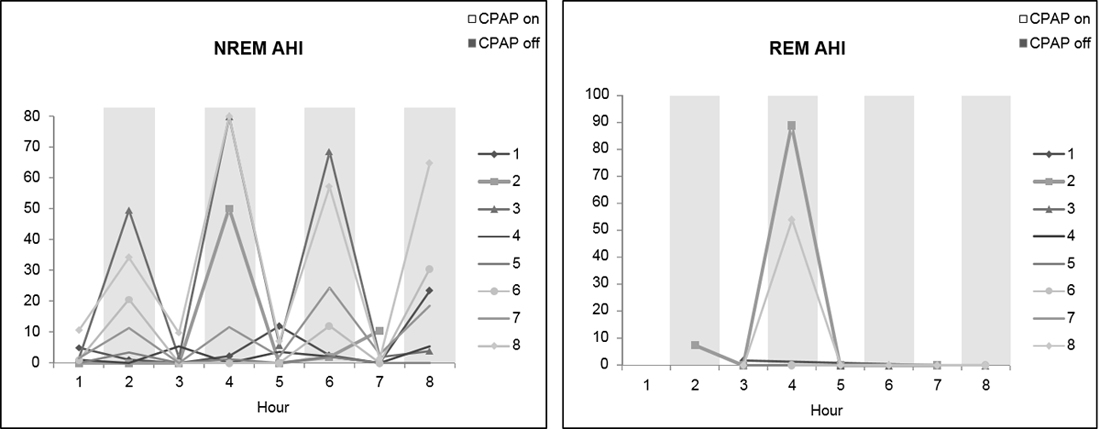

Supplement: S2 Fig — (TIF) [file pone.0146606.s002.tif]
